# Brønsted acid sites based on penta-coordinated aluminum species

**DOI:** 10.1038/ncomms13820

**Published:** 2016-12-15

**Authors:** Zichun Wang, Yijiao Jiang, Olivier Lafon, Julien Trébosc, Kyung Duk Kim, Catherine Stampfl, Alfons Baiker, Jean-Paul Amoureux, Jun Huang

**Affiliations:** 1 Laboratory for Catalysis Engineering, School of Chemical and Biomolecular Engineering, Sydney University, Chemical Engineering Building J01, Sydney, New South Wales 2006, Australia; 2 Department of Engineering, Macquarie University, Sydney, New South Wales 2109, Australia; 3 Univ. Lille, CNRS, UMR 8181-UCCS, Unité de Catalyse et de Chimie du Solide, F-59000 Lille, France; 4 School of Physics, Sydney University, Sydney, New South Wales 2006, Australia; 5 Institute for Chemical and Bioengineering, Department of Chemistry and Applied Bioscience, ETH Zürich, Hönggerberg, HCI, CH-8093 Zürich, Switzerland

## Abstract

Zeolites and amorphous silica-alumina (ASA), which both provide Brønsted acid sites (BASs), are the most extensively used solid acid catalysts in the chemical industry. It is widely believed that BASs consist only of tetra-coordinated aluminum sites (Al^IV^) with bridging OH groups in zeolites or nearby silanols on ASA surfaces. Here we report the direct observation in ASA of a new type of BAS based on penta-coordinated aluminum species (Al^V^) by ^27^Al-{^1^H} dipolar-mediated correlation two-dimensional NMR experiments at high magnetic field under magic-angle spinning. Both BAS-Al^IV^ and -Al^V^ show a similar acidity to protonate probe molecular ammonia. The quantitative evaluation of ^1^H and ^27^Al sites demonstrates that BAS-Al^V^ co-exists with BAS-Al^IV^ rather than replaces it, which opens new avenues for strongly enhancing the acidity of these popular solid acids.

## Abstract (web summary)

Until now, it has been believed that Brønsted acid sites in amorphous silica-alumina are formed from only tetra-coordinated (Al^IV^) sites. Here, the authors use ^27^Al-{^1^H} correlation NMR experiments to identify a new Al^V^-based Brønsted acid site, with implications for increasing the acidity of solid acid catalysts.

The need for efficient and environmentally benign chemical processes has forced the replacement of harmful and corrosive liquid acids by solid acids in various fields of catalysis, including fine chemistry^1^,^2^,^3^, renewable energy production^4^,^5^,^6^, oil refining and petrochemical industries^7^,^8^. Silicon- and aluminum-based mixed oxides provide moderate and strong Brønsted acidity and are among the most popular solid acids used in current chemical processes^7^,^9^. Briefly, the solid acid catalysts can protonate hydrocarbon molecules to form carbocations and drive important reactions, such as cracking, hydrocracking, isomerization, alkylation and aromatization^10^,^11^,^12^,^13^,^14^, through surface complexes or transition states^15^.

Crystalline zeolites and amorphous silica-alumina (ASA) are two main types of solid acids that contain Brønsted acid sites (BASs). It has been widely believed that only tetra-coordinated aluminum (Al^IV^) atoms are able to contribute to the formation of BASs in nature^16^. In crystalline zeolites, the BASs are formed by protons, which compensate the negatively charged oxygens induced by the substitution of Si atoms by Al^IV^ in the framework. The structure of these sites is well known as the bridging Si(OH)Al^IV^ model (Fig. 1a)^15^,^16^,^17^. Replacing Si atoms by more Al^IV^ species can enhance the density of BASs, but it reduces the mean electronegativity of the framework, which thus leads to a decrease of the overall acid strength of BASs^18^,^19^. Similarly, Al^IV^ species incorporated into the amorphous silica network are able to generate BASs on ASA^9^,^20^,^21^. The proximity between Al^IV^ and silanol sites in ASA has recently been observed by nuclear magnetic resonance (NMR) correlation experiments between ^29^Si and ^27^Al nuclei, the sensitivity of which was enhanced by dynamic nuclear polarization^22^. However, the strength of these BASs is generally lower than that on crystalline zeolites^7^ and thus the presence of bridging OH groups (Fig. 1a) in ASA is still strongly under debate^22^,^23^.

A flexible coordination between the Al^IV^ atom and the neighbouring silanol oxygen atom (Fig. 1b)^9^,^21^,^24^,^25^ or a pseudobridging silanol (PBS) with a nearby Al atom (Fig. 1c)^26^,^27^ have been proposed^22^, to account for the longer Al-O distances (2.94–4.43 Å) in ASA^26^, with respect to those in the crystalline zeolite framework (1.88–2.0 Å)^28^. So far, most efforts focus on tuning the concentration of Al^IV^ as the main route to increase the Brønsted acidity on zeolites or silica-alumina^29^,^30^,^31^,^32^,^33^. However, Al^IV^ species tend to condense, to form an alumina phase at high Al/Si ratios^34^,^35^,^36^, leading to the decrease of Brønsted acidity. For ASA containing solely BASs based on Al^IV^ species (BAS-Al^IV^), the maximum Brønsted acidity has been obtained at 30 wt% Al loading^37^,^38^. In spite of the different BAS models, only Al^IV^ species have been experimentally confirmed to contribute to the formation of BASs in these catalysts. Al^V^ and Al^VI^ species have been shown to act as Lewis acid sites on ASA and zeolites, but, to the best of our knowledge, no experimental evidence of BASs involving these sites has been reported so far^39^,^40^,^41^,^42^.

Herein, we provide the direct experimental evidence for a new type of BAS-Al^V^ in ASA by dipolar-mediated heteronuclear multiple quantum correlation (*D*-HMQC) two-dimensional (2D) NMR experiments, which allow the detection of protons via ^27^Al nuclei, hereafter noted ^27^Al-{^1^H}, hence probing the spatial proximities between different Al species and surface protons^43^,^44^,^45^. These experiments show that ASA can contain a large amount of Al^V^ species located near SiOH groups. The acidity of these surface BAS-Al^V^ sites has been demonstrated in this research by the adsorption of basic ammonia molecules, which react with BAS-Al^V^ to form surface ammonium ions.

## Results

### Probing the connectivity between Al^V^ species and SiOH groups

The ASAs used in this work (see Supplementary Methods) have been prepared according to a previously described procedure^9^, which generates ASA nanoparticles with a large amount of Al^V^ species. The ASA powders are designated as SA/*X*, where *X* is 10 or 50, indicating the molar fraction of Al in the precursor with respect to the total amount of Al and Si atoms. The obtained ASAs have tunable BAS acidity strengths ranging from moderate (SA/10 has an acidity close to zeolite H-X) to large (SA/30–70 have stronger BASs than zeolites H-Y and ZSM-5), depending on the aluminum content, as confirmed by both ^13^C magic angle spinning (MAS) NMR investigation with probe molecule acetone and ammonia-temperature program desorption (TPD)^9^. The ASAs exhibited excellent catalytic performances for the conversion of phenylglyoxal with various alcohols, better than that of dealuminated zeolite Y, which hitherto was considered to be the most active solid acid in phenylglyoxal conversion^2^.

**Figure 1:**
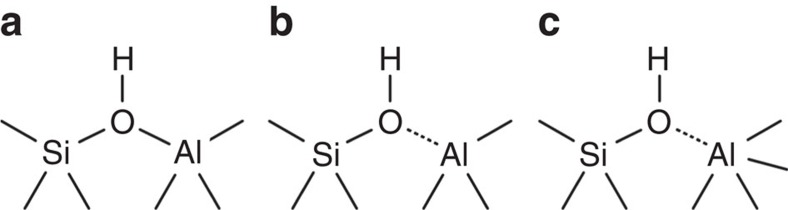
Proposed models for BASs in silica-alumina catalysts. (**a**) BAS consisting of a bridging silanol site bonded to Al^IV^ site (Si(OH)Al) in zeolites^15^. (**b**) BAS consisting of the flexible coordination between silanol oxygen and neighbouring Al^IV^ (ref. 24). (**c**) BAS consisting of PBS interacting with Al^IV^ site^27^. In that later case, the dotted line does not denote a covalent bond but only the close proximity between O and Al atoms.

**Figure 2:**
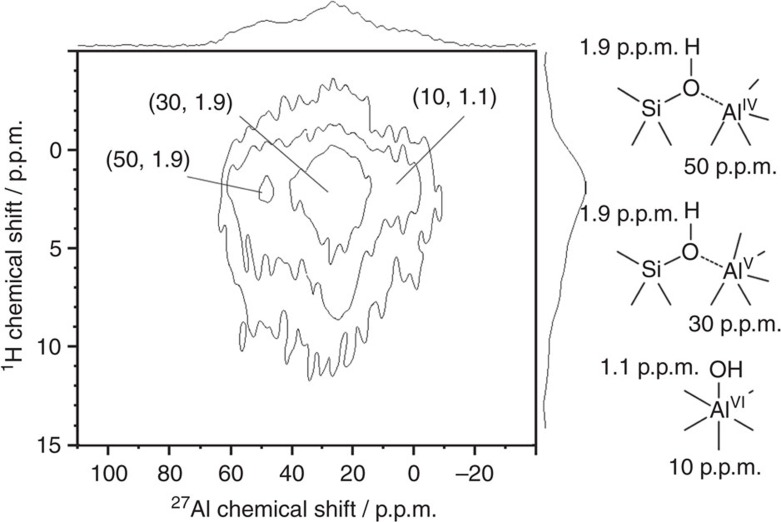
^27^**Al-{**^**1**^**H}** ***D*****-HMQC 2D spectrum of SA/50**. The sample was dehydrated at 723 K for 12 h under vacuum and recorded at 18.8 T with a MAS frequency of *ν*_R_=20 kHz and *τ*_rec_=1.0 ms. The spectrum reveals that the proximity between SiOH groups and Al^V^ species is dominant.

The formation of BAS requires the aluminum atoms to be close to SiOH groups. Such proximity induces a dipolar coupling between ^27^Al and ^1^H nuclei, which can efficiently be probed by *D*-HMQC NMR 2D experiments based on coherence transfers via the ^1^H-^27^Al dipolar couplings^46^,^47^. As shown in Fig. 2, the correlation at *δ*_27A_=50 p.p.m. and *δ*_1H_=1.9 p.p.m. in the ^27^Al-{^1^H} *D*-HMQC 2D spectrum of dehydrated SA/50 indicates a close proximity between Al^IV^ species and the proton of SiOH groups. This correlation is ascribed to the Si-OH···Al^IV^ coordination: the typical BAS-Al^IV^ often described for ASA (Fig. 1c)^16^. A very weak correlation at *δ*_27Al_=10 p.p.m. and *δ*_1H_=1.1 p.p.m. is assigned to the non-acidic terminal Al^VI^OH groups often observed on the surface of silica-alumina or zeolites, whereas the low-field broad hump at *ca*. 6 p.p.m. in the ^1^H dimension could be caused by the small fraction of hydrogen-bonded AlOH groups^16^. Nevertheless, the most intense correlation is observed between Al^V^ species (*δ*_27A_=30 p.p.m.) and SiOH protons (*δ*_1H_=1.9 p.p.m.), which indicates the close proximity between SiOH groups and Al^V^ species, and the presence of Si-OH···Al^V^ coordination (Fig. 2) in dehydrated SA/50.

### Al^V^-based BASs

In zeolites, the substitution of a framework Si atom by an Al^IV^ one to form one SiOHAl acid site (Fig. 1a) can shift the ^1^H NMR signal of SiOH from *ca*. 1.8 to 3.6–5.2 p.p.m. (ref. 16). For these catalysts, BAS could be directly evidenced by the cross-peak in ^27^Al-{^1^H} *D*-HMQC 2D spectrum between Al^IV^ species (*δ*_27Al_=60 p.p.m.) and the bridging OH groups (*δ*_1H_=4.3 p.p.m.)^43^. However, previous works have shown that the Al atoms with neighbouring SiOH groups (Fig. 1b,c) do not produce such a shift of the ^1^H MAS signal of these groups^5^,^6^,^9^,^16^,^20^,^21^. Supplementary Fig. 1a,b show that the ^1^H signal of SA/10 and SA/50 is centred around 1.9 p.p.m., thus indicating a majority of flexible or PBS coordination rather than zeolitic bridging coordination between SiOH groups and either Al^IV^ or Al^V^ species.

Experiments using probe molecules have confirmed the role of flexible or PBS Si-OH···Al^IV^ coordination as BAS in ASA^5^,^6^,^9^,^16^,^20^,^21^. Similar methods using ammonia probe molecules were applied here to demonstrate the acidity of the Si-OH···Al^V^ coordination observed in dehydrated SA/10 and SA/50 (ref. 16). For these samples loaded with ammonia, the ^1^H signal of ammonium ions was observed at *δ*_1H_=6.7 p.p.m., as shown in Supplementary Fig. 1c,d, and commented in Supplementary Note 1. The formation of these ions shows that ammonia reacts with BAS of SA/10 and SA/50.

**Figure 3:**
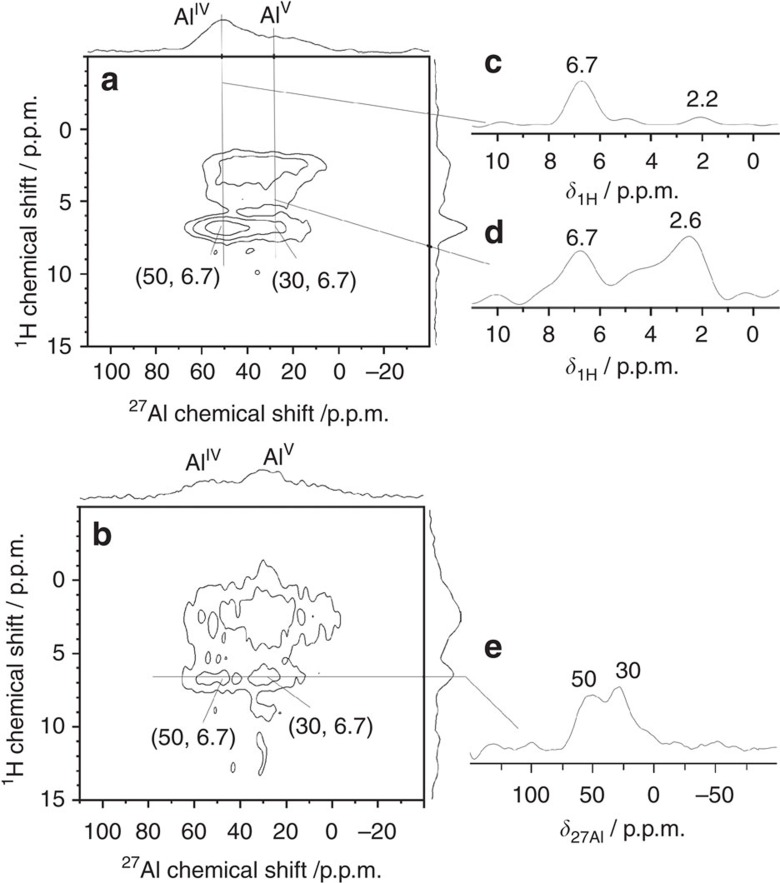
^27^**Al-{**^**1**^**H}** ***D*****-HMQC 2D spectra of ammonia-loaded ASA samples.** The dehydrated SA/10 (**a**) and SA/50 (**b**) samples were loaded with ammonia and evacuated at 373 K for 1 h, and the spectra were recorded at 18.8 T with *ν*_R_=20 kHz and *τ*_rec_=900 μs. The ^1^H slices at the shifts of Al^IV^ and Al^V^ sites of SA/10 extracted from the 2D spectrum (**a**) are displayed in subfigures (**c**,**d**), respectively. The subfigure (**e**) shows the ^27^Al slice at the shift of NH_4_^+^ protons in SA/50 extracted from the spectrum (**b**).

**Figure 4:**
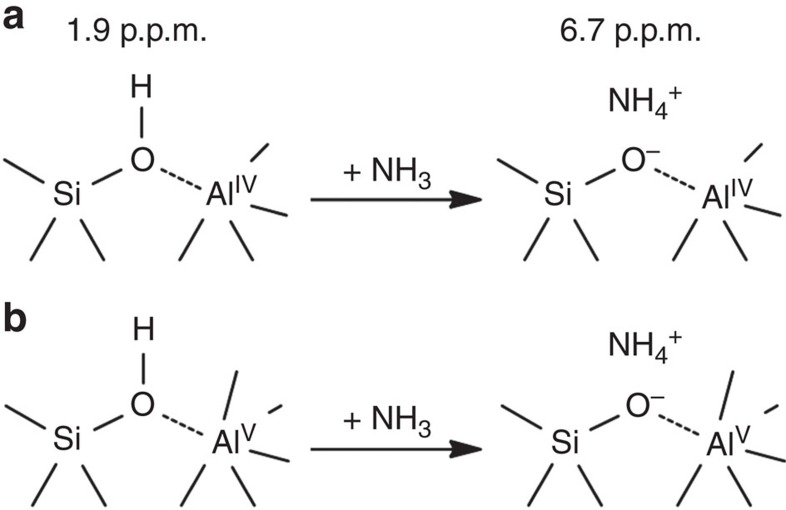
Proposed proton transfer between BAS and ammonia molecules. (**a**) Ammonia protonated on conventional acidic BAS-Al^IV^. (**b**) BAS-Al^V^ formed on ASA is able to transfer hydroxyl proton to ammonia, showing similar acidic properties as BAS-Al^IV^.

^27^Al-{^1^H} *D*-HMQC experiments were also carried out to determine the nature of BAS, which protonate the ammonia molecules. Such a strategy based on ^1^H-^27^Al correlations has been applied for [Al]MCM-41 loaded with ammonia. For such catalysts, ammonium ions (*δ*_1H_=6.7 p.p.m.) were only coupled to Al^IV^ species (*δ*_27A_=56 p.p.m.)^44^. Hence, there was only evidence for BAS-Al^IV^ on the surface of [Al]MCM-41, which protonated ammonia to ammonium ions. As seen in Fig. 3, a correlation between NH_4_^+^ ions (*δ*_1H_=6.7 p.p.m.) and Al^IV^ (*δ*_27Al_=50 p.p.m.) is also observed in ^27^Al-{^1^H} *D*-HMQC spectra of SA/10 and SA/50, showing that the BAS-Al^IV^ sites are also present on the surface of ASAs (Fig. 4a). Interestingly, these spectra also exhibit cross-peaks between Al^V^ species (*δ*_27Al_=30 p.p.m.) and NH_4_^+^ ions (*δ*_1H_=6.7 p.p.m.) in both SA/10 and SA/50. As seen in Fig. 3e, the intensity of this Al^V^-NH_4_^+^ cross-peak is comparable to that of the Al^IV^-NH_4_^+^ one. Given the BAS density ranging from 0.16 to 0.36 H^+^ nm^−2^ in the investigated ASA samples (Supplementary Table 1), each ammonia molecule only interacts with one BAS. The distance between the aluminum atom and the neighbouring silanol oxygen in ASA ranges from *ca*. 2.94 to 4.43 Å^26^ and the N–H bond length in ammonia is only 1.02 Å^48^. As the heteronuclear coherence transfer in ^27^Al-{^1^H} *D*-HMQC is only effective up to a few angstroms, the protons of Si-O^−^(NH_4_)^+^···Al environment only interact with the neighbouring Al. The observation of an Al^V^-NH_4_^+^ cross-peak in Fig. 3 at (30, 6.7) p.p.m. directly confirmed that ammonia is protonated on a BAS containing Al^V^: the Si-OH···Al^V^ group.

The comparison of Supplementary Fig. 1c,d shows that more ammonia molecules are protonated on BAS-Al^V^ in SA/50 than in SA/10. Combined with quantitative ^1^H NMR investigations (Supplementary Fig. 1) and the quadrupolar parameters (Supplementary Table 2 and Supplementary Note 2) obtained from ^27^Al one-dimensional MAS (Supplementary Fig. 3) and 2D multiple quantum MAS (Supplementary Fig. 2) NMR experiments, the analysis of ^27^Al cross-peak intensities in ^27^Al-{^1^H} *D*-HMQC spectra (Supplementary Fig. 4 and Supplementary Note 3) revealed that the population densities of both BAS-Al^IV^ and -Al^V^ on SA/50 (0.078 and 0.053 mmol g^−1^) were both higher than those of SA/10 (0.058 and 0.039 mmol g^−1^). This result suggests that BAS-Al^IV^ and -Al^V^ can co-exist on the surface rather than replacing each other and the population of both acid sites can be amplified by increasing Al content. Thus, this observation is promising for enhancing the population of BAS on ASA without limitation imposed by the Al contents. It should be noted that the **F**_**2**_ projections of ^27^Al-{^1^H} *D*-HMQC 2D spectra are almost identical for dehydrated and ammonia-loaded SA/50 (see Supplementary Fig. 5 and Supplementary Note 4). Therefore, Si-OH···Al^V^ and Si-OH···Al^IV^ coordinations remained unchanged after the protonation of ammonia (as shown in Fig. 4). No Si-OH···Al^IV^ have been transferred to Si-OH-Al^V^ permanently after the adsorption of ammonia. In other words, the NMR results do not show the formation of permanent covalent bridges between silicate and Al^IV^ or Al^V^ sites in ASA samples after the deprotonation of BAS reacting with ammonia. Ammonia partially interacting with surface Al^IV^ or Al^V^ sites (Fig. 3b and Supplementary Fig. 6) was also observed, which has been assigned to ammonia adsorbed on Lewis sites (Supplementary Note 5).

As shown in Fig. 1, a surface bridging SiOHAl (Fig. 1a), a flexible coordination of SiOH and Al (Fig. 1b), or a pseudo-bridge between SiOH and Al atom (Fig. 1c) have been proposed for the formation of BAS-Al^IV^ on ASAs. By analogy, similar structures might also contribute to the formation of BAS-Al^V^. The PBS model permits an explanation of the observation of the ^1^H NMR signal of SiOH at 1.9 p.p.m. in both Fig. 2 and Supplementary Fig. 1, whereas this ^1^H signal of bridging OH groups (Fig. 1a) should occur at 3.6–5.2 p.p.m. If bridging OH groups are present in the investigated ASAs, their concentration must be below the limit of detection of the one-dimensional NMR MAS spectra of Supplementary Fig. 1. Nevertheless, the current NMR data cannot rule out, in addition to PBS, the presence of bridging silanol groups in low concentration in ASA samples. These elusive strong BASs may also contribute to the catalytic activity in spite of their low concentration. The identification of all catalytic BASs in ASAs is beyond the scope of the present study, which is mainly to report the existence of BASs based on Al^V^ environments. A final assessment of the local structure of BAS-Al^V^ will require further experimental and theoretical work.

In summary, a new type of BAS-Al^V^ has been directly observed by ^27^Al-{^1^H} *D*-HMQC NMR spectroscopy. Hitherto, it was widely accepted that Al^V^ sites only provide Lewis acidity^39^,^40^,^41^,^42^, and that solely Al^IV^ ones contribute to the formation of BASs in aluminosilicate. However, we prove here by NMR experiments that similar to Al^IV^ sites, Al^V^ ones interact with neighbouring SiOH groups in ASA and behave as BASs in agreement with the PBS model. BAS-Al^IV^ and -Al^V^ seem to be structurally similar and show comparable acidity to protonate ammonia. Finally, a very important implication emerging from this work is that both Al^IV^ and Al^V^ species can co-exist on the surface of ASA. This feature facilitates that the total population density of BAS can be increased up to 70% by increasing the Al content, an amount much higher than the maximum Al loading of *ca*. 30% at which maximum acidity on ASA containing exclusively BAS-Al^IV^ is achieved^37^,^38^. Hence, our findings not only report the existence of a new type of BAS in nature, but also open new avenues for creating high-performance solid acid catalysts containing Al^V^ species, which will be promising for sustainable oil-refining and many industrial chemical processes.

## Methods

### ^27^Al-{^1^H} *D*-HMQC 2D experiment

All NMR experiments were recorded on a Bruker Avance III 18.8 T (^1^H Larmor frequency of 800 MHz) spectrometer equipped with a 3.2 mm double-resonance MAS probe, in which rotors were spun at *ν*_R_=20 kHz. In the *D*-HMQC sequence, we have detected the ^27^Al nuclei to benefit from their fast longitudinal relaxation times and the ^1^H-^27^Al dipolar couplings were reintroduced by applying a SR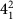 recoupling on the ^1^H channel^49^. The ^1^H radiofrequency amplitudes for the 90° pulses and the SR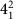 recoupling were equal to *ν*_1_=62.5 and 40 kHz, respectively. The central transition selective pulse lengths on ^27^Al were 8 and 16 μs for 90° and 180° pulses, respectively, that is, radiofrequency field amplitude *ν*_1_=10 kHz. The total dipolar recoupling time, *τ*_rec_, ranged from 700 to 1,000 μs depending on the sample. The 2D spectra resulted from the accumulation of 512 transients for each of 20 *t*_1_ increments with Δ*t*_1_=50 μs and a recycle delay=1 s, that is, a total experiment time of about 3 h. Additional details about NMR experiments are given in the Supplementary Methods.

### Data availability

The data that support the findings of this study are available upon request from the corresponding author J.H. and J.-P.A.

## Additional information

**How to cite this article:** Wang, Z. *et al*. Brønsted acid sites based on penta-coordinated aluminum species. *Nat. Commun.* **7,** 13820 doi: 10.1038/ncomms13820 (2016).

**Publisher's note:** Springer Nature remains neutral with regard to jurisdictional claims in published maps and institutional affiliations.

## Supplementary Material

Supplementary Information Supplementary Figures, Supplementary Tables, Supplementary Methods, Supplementary Notes and Supplementary References.
