# Supplementary material for: Brønsted acid sites based on penta-coordinated aluminum species
Source: Nat Commun. 2016 Dec 15;7:13820. doi: 10.1038/ncomms13820 (PMC5172364; doi:10.1038/ncomms13820)
Supplement: Supplementary Information — Supplementary Figures, Supplementary Tables, Supplementary Methods, Supplementary Notes and Supplementary References. [file ncomms13820-s1.pdf]

Supplementary Figures

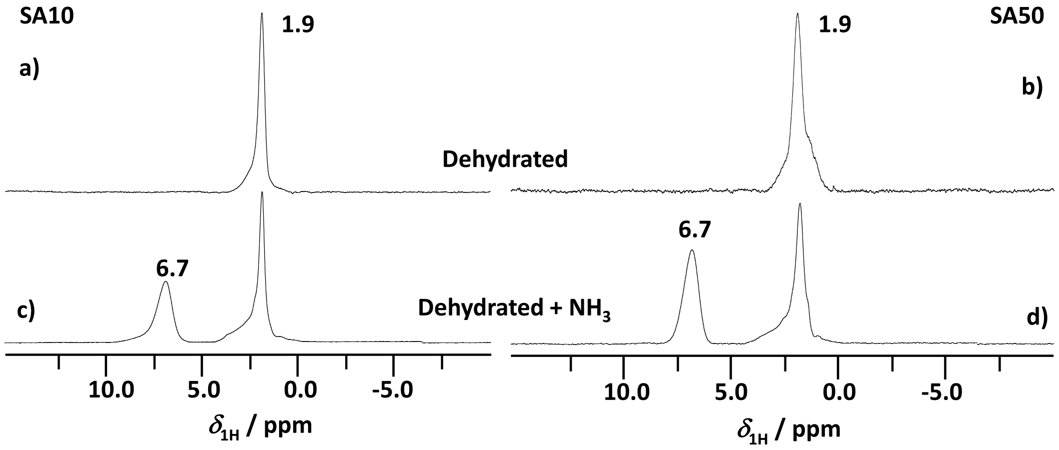

Supplementary Figure 1. ^{1}H MAS NMR spectra of ASA samples. Spectra of SA/10 (a,c) and SA/50 (b,d) dehydrated at 723 K (a,b), and dehydrated and loaded with NH_3 (c,d), respectively, recorded at 18.8 T and ν_R = 20 kHz using DEPTH sequence.

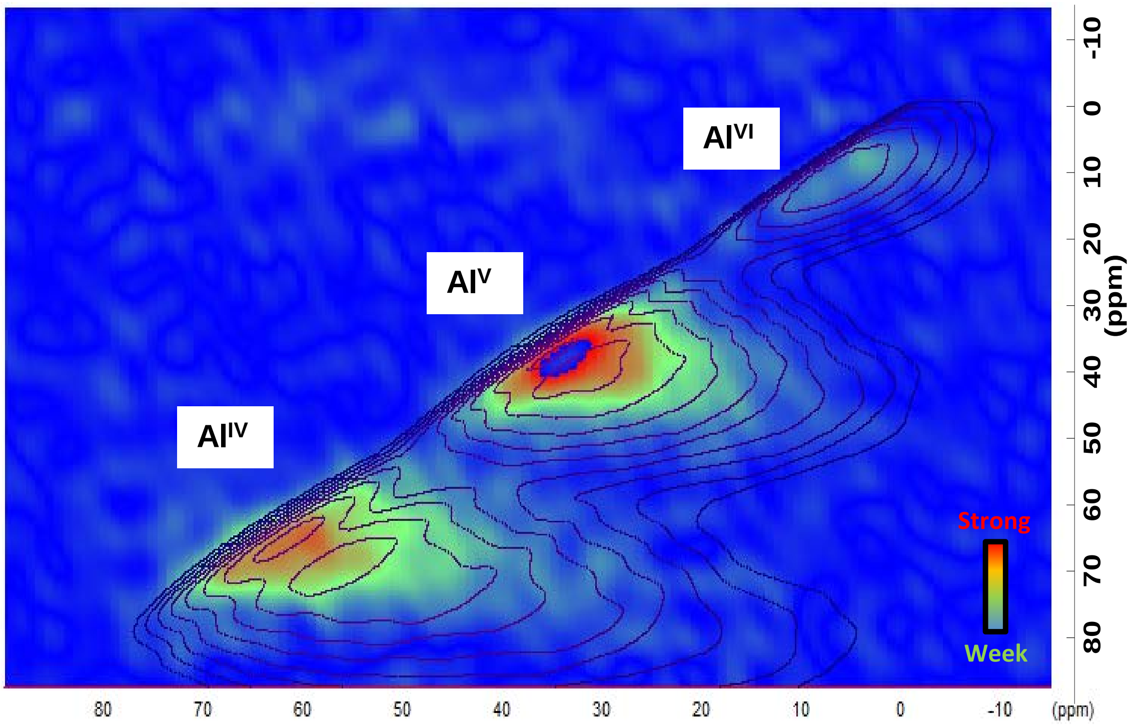

Supplementary Figure 2. ^{27}Al three-pulse 3QMAS 2D NMR spectrum. This spectrum of SA/50 dehydrated at 723 K and loaded with ammonia indicates a distribution of the local environment, which results in a distribution of chemical shifts and C_Q/η_Q values. The simulation (contour lines) is consistent with experiment (color map). The lower parts of each resonance are not observed because they correspond to contributions with large quadrupole constants (C_Q > 15 MHz), which are not properly excited by MQMAS with this moderate value of ν_1 = 100 kHz. This simulation has been done with the same parameters and intensities as determined from the fit of the 1D spectrum (see Supplementary Fig. 3a).

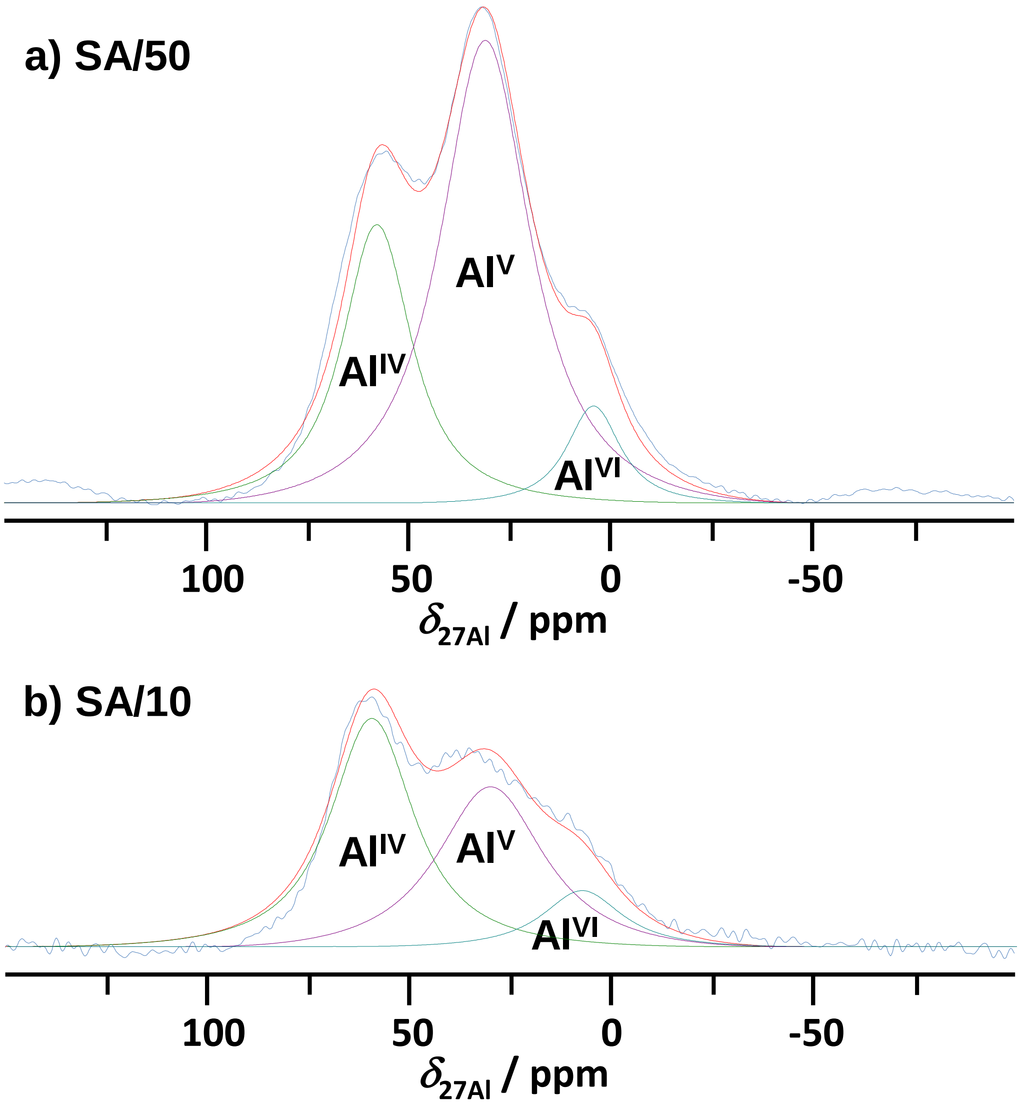

Supplementary Figure 3. ^{27}Al direct excitation MAS spectra of dehydrated ASA samples. a) SA/50 and b) SA/10 samples were dehydrated at 723 K and loaded with ammonia, and recorded at 18.8 T and ν_R = 20 kHz. The pulse length was t_p = 1 µs and the rf field strength was ν_1 = 100 kHz. These parameter values allow the acquisition of ^{27}Al signals under quantitative conditions and would produce a flip angle of 30° for ^{27}Al nuclei in an isotropic liquid. *NS* = 2048 transients with *RD* = 0.5 s and *NS* = 640 with *RD* = 4 s were accumulated for SA/10 and for SA/50, respectively. Longitudinal relaxation T_1 was 0.5 and 1.5 s for SA/10 and SA/50, respectively.

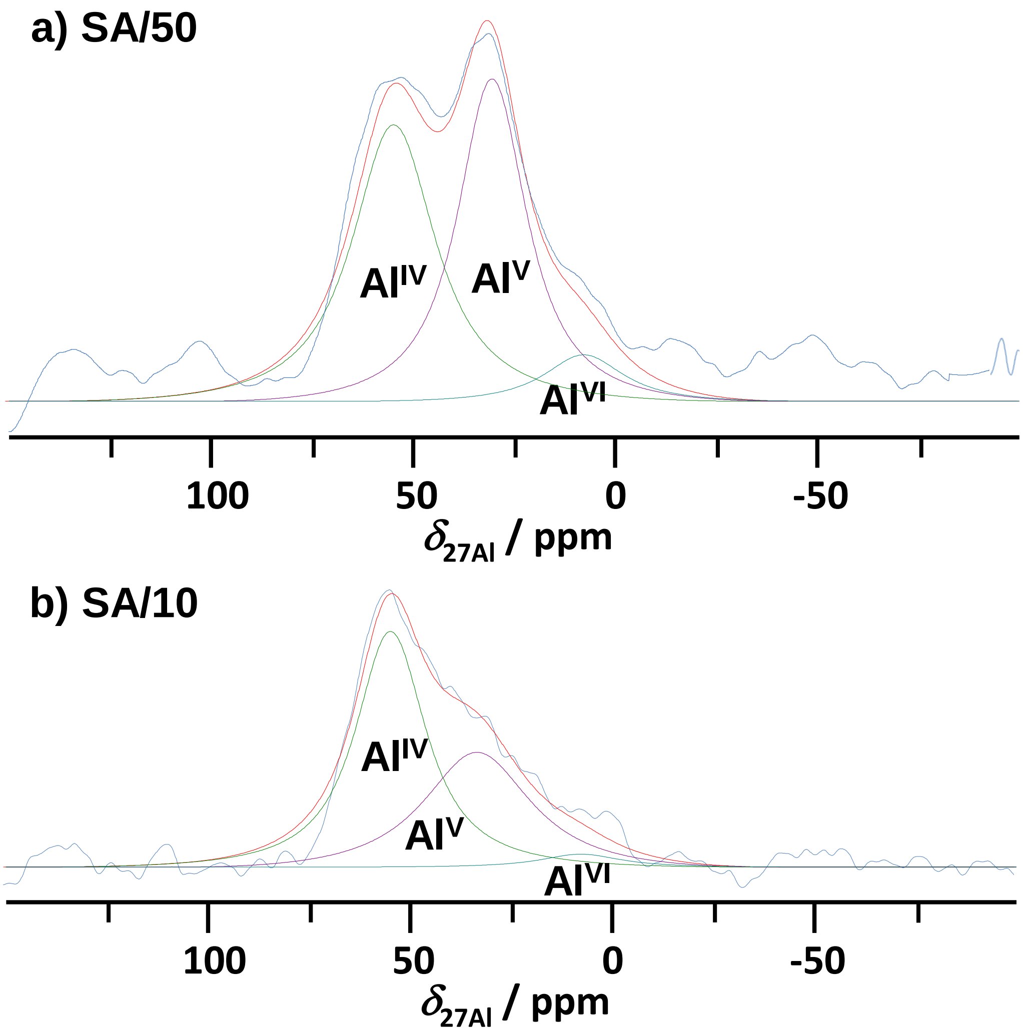

Supplementary Figure 4. Deconvolution of the ^{27}Al slices. The slices were obtained (sum of 5 rows from δ_{1H} = 6.3 to 7.2 ppm) from the ^{27}Al-{^{1}H} *D*-HMQC spectra shown in Fig. 2 of SA/10 and SA/50 dehydrated at 723 K and loaded with ammonia. The deconvolution parameters are listed in Supplementary Table 2. As the C_Q values, the number of neighboring protons and the Al-H distances for Al^{IV} and Al^{V} sites are similar, these slices can be utilized to estimate the relative amount of these two ^{27}Al environments.

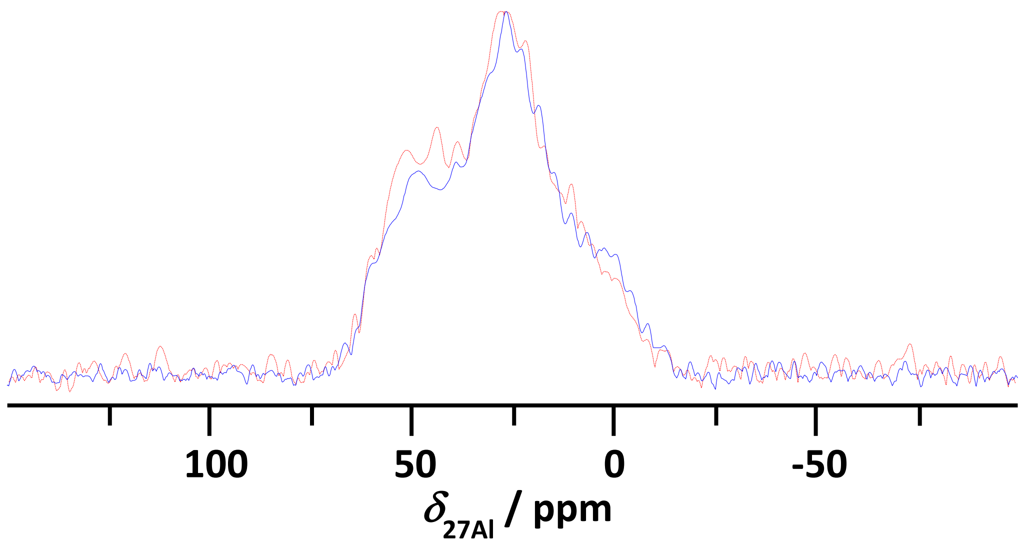

Supplementary Figure 5. The F_2 projections of ^{27}Al-{^{1}H} *D*-HMQC spectra. The spectra of dehydrated (blue line) and ammonia-loaded (red line) SA/50 acquired for the same amount of sample.

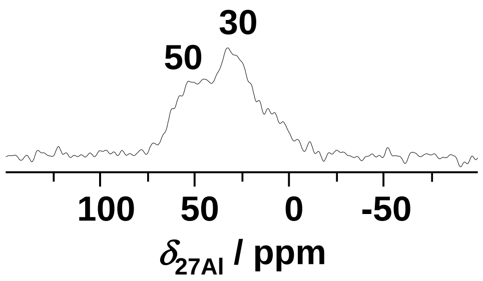

Supplementary Figure 6. Trace of Fig. 3b at δ_{1H} = 2.4 ppm.

Supplementary Tables

Supplementary Table 1. The specific surface areas, the concentration and the density of OH groups and BAS of these ASA materials.

	A^{a} $m^2.g^{-1}$	Concentration of OH groups[a] mmol.g^{-1}	Concentration of BAS[a] mmol.g^{-1}	Density of OH[b] nm^{-2}	Density of BAS[b] nm^{-2}
SA/10	377	1.273	0.098	2.03	0.16
SA/50	222	1.055	0.134	2.86	0.36

[a] The surface areas (*A*) and the concentration of OH groups and BAS of ASA were taken from ref. 1. The particle size for both SA/10 and SA/50 samples was ca. 7-12 nm as shown in SEM images from ref. 1.

[b] The densities of OH and BAS were determined from A_{BET} the specific surface areas and the concentration of OH groups and BAS given in column 1, 2 and 3, respectively.

Supplementary Table 2. Parameters employed for the deconvolution of ^{27}Al 1D MAS spectra of SA/10 and SA/50 dehydrated and loaded with ammonia with the relative fractions of the different ^{27}Al environments determined from ^{27}Al-{^{1}H} *D*-HMQC spectra. The deconvolutions of the spectra are displayed in Supplementary Fig. 3 and 4.

	SA/10, dehydrated + NH_3				SA/50, dehydrated + NH_3			
	δ_{cs}^{av}[a] ppm	C_Q^{avb} MHz	f^c %	density[d] mmol.g^{-1}	δ_{cs}^{av}[a] ppm	C_Q^{avb} MHz	f^c %	density[d] mmol.g^{-1}
BAS-Al^{IV}	64	5.7	58.1	0.057	64	5.7	47.3	0.063
BAS-Al^{V}	36	5	38.6	0.041	36	5	45.7	0.071
Al^{VI}	11	3.8	3.3		7	4.6	7	

[a] Average isotropic chemical shift determined from the deconvolution of ^{27}Al 1D direct excitation MAS spectra (see Supplementary Fig. 3).
[b] Average C_Q constant determined from the deconvolution of ^{27}Al 1D direct excitation MAS spectra (see Supplementary Fig. 3).
[c] Fraction of a given ^{27}Al local environment determined from the deconvolution of ^{27}Al-{^{1}H} *D*-HMQC slice at δ_{1H} = 6.7 ppm (Supplementary Fig. 4).
[d] Density of BAS-Al^{IV} and BAS-Al^{V} sites determined from the density of BAS obtained from quantitative ^{1}H MAS NMR spectra (Supplementary Fig. 1) and the fractions of BAS-Al^{IV} and BAS-Al^{V} measured from the ^{27}Al-{^{1}H} *D*-HMQC slice.

Supplementary Methods

Preparation o f A SA Amorphous silica-alumina (ASA) catalysts were prepared within microseconds at extremely high temperature (ca. 2000 K) by the flame-spray pyrolysis technique.[4] Briefly, the appropriate amounts of the precursor materials were dissolved in a 1:1 (vol.) mixture of acetic acid and methanol. The resulting solution was filtered using a glass filter, pumped through a capillary at a rate of 5 mL.min^{-1}, and nebulized by using an O_2 flow rate of 5 L.min^{-1} and ignited by an annular supporting methane/oxygen flame (1.5/0.9 L.min^{-1}) to generate ASA nanoparticles with a large amount of Al^{V} species. The specific surface areas, the concentration of OH groups and the density of BAS of these ASA materials were determined in our previous works[4] and summarized in Supplementary Table 1. The densities of BAS was 0.16 and 0.36 H^+/nm^2 on SA/10 and SA/50, respectively. Therefore, the average distance between acidic silanols was ca. 45 and 30 Å on SA/10 and SA/50, respectively. As the heteronuclear coherence transfer in ^{27}Al-{^{1}H} *D*-HMQC is only effective up to a few angstroms, a given ^{27}Al nucleus correlates with a single silanol group.

Sample preparation for NMR investigation. Before NMR experiments, the samples filled in glass tubes were dehydrated at 723 K for 12 h at a pressure lower than 10^{-2} bar. These dehydrated samples were sealed in the glass tubes or directly loaded with ammonia on a vacuum line. Subsequently, the loaded samples were evacuated at 393 K for 1 h to remove the weakly physisorbed molecules. The samples were then transferred into the MAS rotors under dry nitrogen gas inside a glove bag. As already mentioned in the main text, the MAS frequency for all NMR experiments was ν_R = 20 kHz.

^{1}H M AS 1 D NMR experiment. The ^{1}H spectra were acquired using the DEPTH pulse sequence, which allowed quantitative measurements because (i) we used echo times shorter than 40 µs, and (ii) spinning sidebands were small because of weak ^{1}H-^{1}H dipolar couplings.

For these experiments, the rf field amplitude, the number of scans and the recycle delay were equal to: ν_1 = 62.5 kHz, *NS* = 32 and *RD* = 5 s.

^{27}Al 1D MAS and 2D MQMAS experiment. The ^{27}Al NMR 1D MAS spectra were acquired under quantitative conditions with direct-excitation. Additional details about these experiments are given in the caption of Supplementary Fig. 3. 1D spectra were processed with linear back prediction over 10 µs (5 complex points) in order to restore a flat baseline. The ^{27}Al Multiple-Quantum MAS (MQMAS) NMR spectra of SA/10 and SA/50 were recorded using the three-pulse z-filter MQMAS pulse sequence.[2] The 2D spectra were sheared with the xfshear program included in the TOPSPIN software. Excitation and reconversion pulses lasted t_p = 4 and 1.2 µs, respectively, with ν_1 = 100 kHz, and the central-transition (CT) selective $\pi/2$ last pulse was 12 µs with ν_1 = 7 kHz. The 2D spectra resulted from the accumulation of *NS* = 576/192 transients for each of the 8/32 t_1 increments with States-TPPI acquisition, Δt_1 = 25 µs, with a recycle delay of *RD* = 0.5/1.5 s, for SA/10 and SA/50, respectively.

Supplementary Note 1

The ^{1}H MAS NMR spectra of dehydrated SA/10 and SA/50 (Supplementary Fig. 1a and b) are dominated by a strong signal of SiOH groups (δ_{1H} = 1.9 ppm), which masks that of BAS. After loading with ammonia, BAS protonates adsorbed ammonia to ammonium ions, leading to a strong signal at δ_{1H} = 6.7 ppm (Supplementary Fig. 1c and d). It evidences the presence of BAS in ASA materials under study. Moreover, the densities of BAS in SA/10 and SA/50 (9.7 and 13.1 mmol.g^{-1}) have been determined from the integrated intensity of ammonium signals.

Supplementary Note 2

We estimated the quadrupolar parameters of Al^{IV} and Al^{V} nuclei in ASA loaded with ammonia from the ^{27}Al MQMAS NMR 2D spectra (Supplementary Fig. 2) and the direct excitation ^{27}Al 1D spectrum acquired under quantitative conditions.[3,5] Based on previous work,[6] the resonance frequencies along both spectral dimensions of the ^{27}Al MQMAS signal allow the calculation of the second-order quadrupolar effect (SOQE) parameter according to

$$SOQE^2 = \delta_{\mathrm{CS}} - \delta_{\mathrm{F2}}k \quad (1)$$

where the isotropic chemical shift δ_{cs} is:

$$\delta_{\mathrm{cs}} = \frac{17}{27}\delta_{\mathrm{F1}} + \frac{10}{27}\delta_{\mathrm{F2}} \quad (2)$$

δ_{F1} and δ_{F2} are the gravity centers of the signals in the **F$_1$** and **F$_2$** dimension, and

$$k = \frac{3}{10}\frac{4I(I+1)-3}{[4I(2I-1)v_0]^2} \times 10^6 \quad (3)$$

where I = 5/2 is the spin quantum number of ^{27}Al and v_0 its Larmor frequency. Then, the quadrupolar coupling constant can be estimated from the SOQE value since:

$$C_Q = \frac{\mathrm{SOQE}}{\sqrt{1 + (\eta_Q^2/3)}} \qquad (4)$$

where, the range of asymmetry parameters is limited to $0 \leq \eta_Q \leq 1$, and thus the range of the quadrupolar coupling constant is restricted to SOQE $\leq C_Q \leq$ 1.15.SOQE. As a result, the SOQE parameter directly determined from the MQMAS 2D spectra can be used as an initial input C_Q value for the fitting and deconvolution of the ^{27}Al 1D MAS spectra acquired under quantitative conditions (see Supplementary Fig. 3), whereas Supplementary Equation 2 provides an initial input value for the isotropic chemical shift value. The fits of the 1D NMR spectra were carried out using DMfit software[7] using as input parameters those determined from the analysis of the corresponding 2D MQMAS spectrum.[8]

Supplementary Table 2 summarizes the obtained C_Q values and isotropic chemical shift of each Al species obtained from the fit of 1D-MAS ^{27}Al spectra acquired under quantitative conditions. These C_Q values (5-5.7 MHz) are in a good agreement with that of ammonia-loaded amorphous silica-alumina reported in literature (4.6-5.0 MHz).[9,10] The validity of the best-fit parameters reported in Supplementary Table 2 was also checked by simulating the 2D MQMAS spectra (see Supplementary Fig. 2). As expected, the 2D MQMAS experiments filters out the contribution corresponding to the largest C_Q values.

Supplementary Note 3

^{27}Al-{^{1}H} *D*-HMQC 2D MAS spectra

The ^{27}Al 1D spectra subsume the signals of all ^{27}Al nuclei, whether they are BAS or not. Conversely, the ^{27}Al slices of the ^{27}Al-{^{1}H} *D*-HMQC spectra at δ_{1H} = 6.7 ppm correspond to the ^{27}Al sites located in the vicinity of NH_4^+ cations and hence chiefly to the ^{27}Al-BAS. The signal intensities in ^{27}Al-{^{1}H} *D*-HMQC 2D spectra are a priori not quantitative owing to (i) the use of pulses selective for the ^{27}Al central transition and (ii) the ^{1}H-^{27}Al coherence transfers.

However, Al^{IV} and Al^{V} sites have similar C_Q values (see Supplementary Table 2) and similar number of protons in their vicinity (see Fig. 4). Under those conditions, the relative amount of BAS-Al^{IV} and BAS-Al^{V} can be estimated from the relative integrated intensities of the de-convoluted signals of those sites in ^{27}Al-{^{1}H} *D*-HMQC experiment (Fig. 3). The fits shown in Supplementary Fig. 4 used the same parameters determined previously by fitting the 1D MAS (Supplementary Fig. 3), and only the fractions of the aluminum species were allowed to vary. Combined with the densities of BAS (0.097 and 0.131 mmol.g^{-1} for SA/10 and SA/50) determined from Supplementary Fig. 1, the population of BAS-Al^{IV} and BAS-Al^{V} are listed in Supplementary Table 2. Clearly, when increasing Al content from 10 to 50 %, (i) the density of BAS-Al^{V} increases from 0.041 to 0.071 mmol.g^{-1}, but (ii) that of BAS-Al^{IV} also increases from 0.057 to 0.063 mmol.g^{-1}. Obviously, on ASA samples the formation of BAS-Al^{V} does not replace that of BAS-Al^{IV}, and both BAS-Al^{IV} and BAS-Al^{V} co-exist on the surface.

Supplementary Note 4

The $\mathbf{F_2}$ projections of ^{27}Al-{^{1}H} *D*-HMQC spectra of dehydrated (blue line) and ammonia-loaded (red line) SA/50 are shown in Supplementary Figure 5. The two spectra corresponding to the same amount of samples before and after ammonia loading are almost identical, which indicates that Al coordination is unchanged before and after ammonia loading on samples. Therefore, Si-OH···Al^{V} and Si-OH···Al^{IV} coordinations remained the same after the protonation of ammonia. No Si-OH···Al^{IV} has been transferred to Si-OH-Al^{V} permanently after the adsorption of ammonia. The Al^{V}-NH_4^+ cross-peak (30, 6.7) ppm in Fig. 3 was directly from the protonated ammonia on Si-OH···Al^{V} BAS (Si-$O^-$$(NH_4)^+$···$Al^{V}$) rather than bridging Si-$O^-$$(NH_4)^+$-$Al^{V}$ (transferred from Si-OH···Al^{IV} BAS). These NMR results indicate that no permanent bridge between Al site and silanol is formed after the deprotonation of BAS via the adsorption of ammonia.

Supplementary Note 5

NH_3 adsorbed on Lewis acidic Al sites could cause ^{1}H NMR signal in the range of δ_{1H} = 2.6-4.6 ppm.[11,12] The ASA samples had been dehydrated under vacuum at 723 K overnight before ammonia adsorption. Under higher dehydration temperature, part of surface OH groups should be removed and part of surface Al centers could act as Lewis acid sites. The trace of Fig. 3b at δ_{1H} = 2.4 ppm has been shown in Supplementary Figure 6. Some surface Al^{IV} and Al^{V} species have been observed as Lewis acid sites. The ratio between them was proportional to the ratio of BAS-Al^{IV} to BAS-Al^{V}.

Supplementary References

1. Wang, Z., Jiang, Y., Baiker, A. & Huang, J. Efficient acid-catalyzed conversion of phenylglyoxal to mandelates on flame-derived silica/alumina. *ACS Catalysis* **3**, 1573-1577 (2013).
2. Amoureux, J.-P., Fernandez, C. & Steuernagel, S. Z-filtering in MQMAS NMR. *J. Magn. Reson.*, **123**, 116-118 (1996).
3. Guo, Z. *et al*. Pt Nanoclusters Confined within metal-organic framework cavities for chemoselective cinnamaldehyde hydrogenation. *ACS Catal.* **4**, 1340-1348 (2014).
4. Huang, J., van Vegten, N., Jiang, Y., Hunger, M. & Baiker, A. Increasing the Brønsted acidity of flame-derived silica/alumina up to zeolitic strength. *Angew. Chem.-Int. Ed.* **49**, 7776-7781 (2010).
5. Ganapathy, S. *et al*. Anisotropic chemical shielding, M-site ordering, and characterization of extra-framework cations in ETS-10 studied through MAS/MQ-MAS NMR and molecular modeling techniques. *J. Am. Chem. Soc*. **120**, 4752-4762 (1998).
6. Hunger, M. Zeolite characterization and catalysis - A Tutorial. Ed. Chester, W. Arthur, E.G. Derouane, E. G. 65-105 (Springer, 2010).
7. Hunger, M., Schenk, U., Breuninger, M., Glaser, R. & Weitkamp, J. Characterization of the acid sites in MCM-41-type materials by spectroscopic and catalytic techniques. *Microporous Mesoporous Mater.* **27**, 261-271 (1999).
8. Omegna, A., van Bokhoven, J. A. & Prins, R. Flexible aluminum coordination in alumino-silicates. Structure of zeolite H-USY and amorphous silica-alumina. *J. Phys. Chem. B* **107**, 8854-8860 (2003).
9. Jiao, J., Kanellopoulos, J., Wang, W., Ray, S. S., Foerster, H., Freude, D. & Hunger, M. Characterization of framework and extra-framework aluminum species in non-hydrated zeolites Y by Al-27 spin-echo, high-speed MAS, and MQMAS NMR spectroscopy at B-0=9.4 to 17.6 T. *Phys.l Chem. Chem. Phys.* **7** (17), 3221-3226 (2005).

10. Massiot, D. *et al*. Modelling one- and two-dimensional solid-state NMR spectra. *Magn. Reson. Chem.* **40,** 70-76 (2002).

11. Yin, F., Blumenfeld, A. L., Gruver, V. & Fripiat, J. J. NH_3 as a Probe Molecule for NMR and IR Study of Zeolite Catalyst Acidity. *J. Phys. Chem. B* **101**, 1824-1830 (1997).

12. Ma, D. *et al*. An investigation of the roles of surface aluminum and acid sites in the zeolite MCM-22. *Chem.-Eur. J.*, 8 (1), 162-170 (2002).
